# PMS2: a potential prognostic protein marker in oral squamous cell carcinoma

**DOI:** 10.4317/medoral.24303

**Published:** 2020-11-28

**Authors:** Jordana Medeiros Lira Decker, Osias Vieira de Oliveira Filho, Milena Oliveira Freitas, Isabelle Joyce de Lima Silva-Fernandes, Thinali Sousa Dantas, Clarissa Sales de Paula Campêlo, Maria do Perpétuo Socorro Saldanha Cunha, Paulo Goberlânio de Barros Silva, Fabrício Bitu Sousa

**Affiliations:** 1 DDS, Master (Dentistry), Department of Dentistry, Unichristus, Fortaleza, Ceará, Brazil; 2 DDS, Master Student (Dentistry), Division of Oral Pathology, Faculty of Pharmacy, Dentistry and Nursing, Federal University of Ceará, Brazil; 3 BIOL, Ceará School of Oncology, Hospital Haroldo Juaçaba, Fortaleza, Ceará, Brazil; 4 DDS, PhD Student (Dentistry), Division of Oral Pathology, Faculty of Pharmacy, Dentistry and Nursing, Federal University of Ceará, Fortaleza, Brazil; 5 Professor, Department of Dentistry, Unichristus, Fortaleza, Ceará, Brazil; 6 MD, Ceará School of Oncology, Hospital Haroldo Juaçaba, Fortaleza, Ceará, Brazil; 7 DDS, PhD, Division of Oral Pathology, Faculty of Pharmacy, Dentistry and Nursing, Federal University of Ceará, Fortaleza, Brazil

## Abstract

**Background:**

An increase in oral squamous cell carcinoma (OSCC) cases was observed despite the reduction in exposure to classic risk factors. Although the exact cause of this trend remains unknown, epigenetic factors could be contributing to an increased occurrence of these tumors. This study aims to assess the influence of PMS2 protein immunoexpression on the prognosis of patients with OSCC.

**Material and Methods:**

This study comprised 76 cases of OSCC treated between 2011 and 2016. Immunohistochemical staining for PMS2 was performed. For evaluation, 10 fields per histological section were photographed at a 400x magnification and positively-stained cells were counted with Image J. Mann-Whitney and Kruskal-Wallis tests were used to compare the immunolabeling pattern with the clinical-pathological and prognostic characteristics. Survival analysis was performed with Chi-square, Long-Rank Mantel-Cox and Cox regression tests (*p*<0.05).

**Results:**

An overexpression of PMS2 was observed in N0/1 tumors and in oral cancers found in unusual locations. In patients ≤60 years of age, high levels of PMS2 ( >60%; *p*=0.041) were associated with low survival (*p*=0.029). In multivariate analysis, surgery combined with chemotherapy (*p*=0.030) and high PMS2 immunoexpression (*p*=0.042) significantly increased the risk of death for ≤60 years old patients.

**Conclusions:**

The findings of this study indicate that PMS2 can be a potential prognostic protein marker in OSCC patients 60 years of age and younger.

** Key words:**Squamous cell carcinoma, mouth neoplasms, mismatch repair endonuclease PMS2, survival.

## Introduction

Oral squamous cell carcinoma (OSCC) is the most common type of head and neck cancer (HNC). The oral carcinogenesis process originates from genetic and epigenetic alterations, resulting in genomic and cellular instability as well as tumor progression (1). The most important risk factors associated with the development of these turmors are alcohol and tobacco consumption (2). An increase in OSCC cases was observed despite the reduction in exposure to these classic risk factors (3), with non-smoker and non-drinker patients developing oral cancer at an increasing rate worldwide (3,4). Although the exact cause of this trend remains unknown, genetic factors, possibly influenced by environmental agents, could be contributing to an increased occurrence of these tumors. Carcinogens and lifestyle factors may favor tumorigenesis through epigenetic mechanisms and, therefore, the study of these factors could shed light on this alarming trend (2).

A strong link between inactivation of DNA mismatch repair (MMR) proteins and oral carcinogenesis has been reported by several authors (5-9). The MMR pathway is responsible for maintaining genomic stability (9) and it is composed of 3 distinct protein subunits: MutSα (MSH2-MSH6), MutSβ (MSH2-MSH3), and MutLα (MLH1-PMS2) (1). Several mutations in the MMR system have been reported, including PMS2 alterations, which appears to have an endonucleolytic role in the DNA repair process (10). PMS2 has also been described as the MMR-protein most associated with the degree of severity of OSCCs (8). A review of several studies investigating colorectal cancer (CRC) suggested that a hypomorphic PMS2 variant may cause early onset of cancer (11). In addition, PMS2 mutation carriers have often been associated with extra-colonic cancers (10).

Deficiency in MMR proteins has been linked to malignant transformation in many cancers (8,11-13). This process occurs as a result of an accumulation of mutations associated with genetic instability, including microsatellite instability (MSI) (14). Several studies have suggested that dysregulation of the MMR pathway via gene overexpression may produce similar effects in genomic instability (1,12,14,15).

Taking into account the important relationship between the MMR pathway and tumor behavior, the aim of this study was to assess the influence of the PMS2 protein on the prognosis of patients with OSCC as well as to correlate these findings with clinical-pathological aspects.

## Material and Methods

- Patient sample and inclusion and exclusion criteria

The inclusion criteria for this study consisted of specimens from patients treated at Haroldo Juaçaba Hospital, between 2011 and 2016, who underwent oral cancer resection without neoadjuvant treatments. The exclusion criteria were incomplete medical records and specimens with insufficient or damaged material for microscopy and tissue micro array (TMA) technique. The medical data were accessed through an electronic patient record system and the formalin-fixed paraffin-embedded blocks were retrieved from the hospital pathology laboratory archive.

A total of 76 formalin-fixed paraffin-embedded specimens were selected and the respective demographic and clinical data of the patients were analyzed. Histological slides of the excisional biopsies were assessed by a pathologist and graded into well-differentiated, moderately or poorly differentiated groups according to the World Health Organization criteria (16). Tumorous areas with highly cellular sections of OSCC were identified for manufacturing the TMA blocks. Additionally, surgical resection margins and lymph node metastasis were used as control and comparative groups, respectively (17).

- Immunohistochemical staining

Immunohistochemical staining for PMS2 was performed using the streptavidin-biotin-peroxidase technique. Paraffin blocks were cut into 4-mm sections and placed on silanized slides. The slides were de-paraffinized in xylene and hydrated in descending grades of ethanol. Heat-induced antigen retrieval was performed with a high pH solution (Dako®, S2367). Sections were then washed with phosphate-buffered saline (PBS) and incubated in 3% hydrogen peroxide for 30 minutes to block the endogenous peroxidase, followed by overnight incubation with primary antibody hPMS2 (Dako®, EP51, 1:100). The next day, sections were washed with PBS before the application of Envision (Dako®, K406189-2, ready-to-use) for 30 minutes. Slides were then washed with PBS for 10 minutes and incubated with 3,3′-diaminobenzidine-tetrachloride (DAB, Dako®, S196730-2) for 5 minutes and subsequently counterstained with Harris's haematoxylin for 10 seconds. Lastly, slides were dehydrated in ethanol, cleared in xylene, and mounted. Positive (colon) and negative staining controls were conducted according to the manufacturer.

- Cell quantification

For the evaluation of PMS2 levels, 10 fields per histological section were photographed at 400x magnification. The images were exported to the ImageJ® software to determine the percentage of immunostained cells (cell counting command). Cancer cells exhibiting brown nuclear staining were considered as a positive immunoreaction for PMS2.

- Statistical analysis

The data were exported to the Statistical Package for the Social Sciences (SPSS) software, version 20.0 adopting a 95% confidence interval. The percentage of immunostained cells was calculated and expressed as mean and standard deviation and analyzed with the Kruskal-Wallis or Mann-Whitney test. Categorical data were expressed as absolute and percentage frequencies and were analyzed using the chi-square and Long-Rank Mantel-Cox. Variables significantly associated with overall survival were assessed with Cox’ multivariate regression model.

## Results

Eighty-six percent (86.8%) of patients remained alive during the five years of evaluation, totaling an average survival of 49.11±25.13 months.

The median age was 59 (range = 23 to 87) years and most patients were ≤60 years old (n=43, 56.6%), male (n=50, 65.8%) and had tumors on the floor of the mouth (n=35, 46.1%). Smoking history was described in 51 (67.1%) cases and alcohol consumption in 21 (27.6%). These factors showed no association with the 5-year survival (Table 1).

**Table 1:**
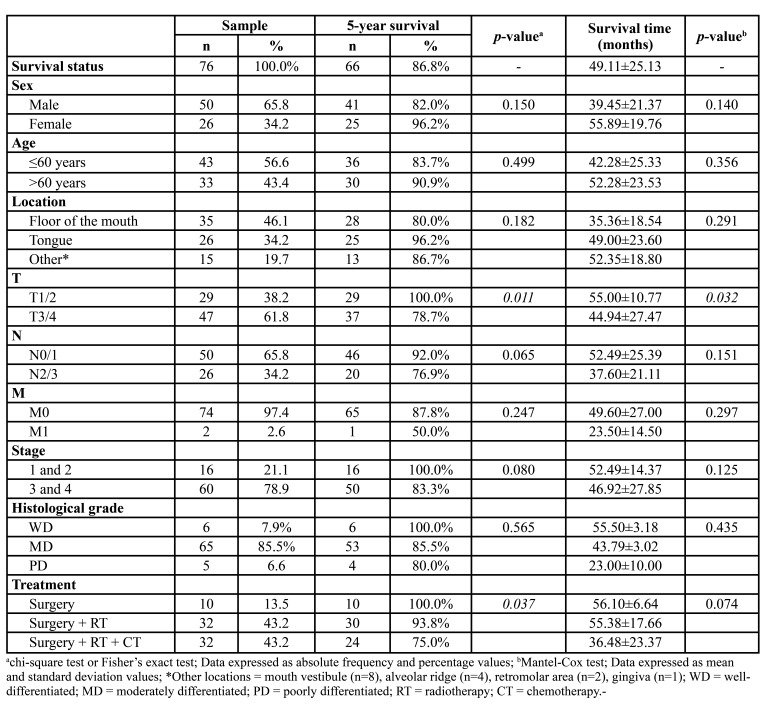
Clinical-epidemiological profile of the selected patients with oral cancer treated at the Hospital Haroldo Juaçaba (Cancer Institute of Ceará) between 2011 and 2016.

T3/4 cases showed lower survival (n=47, 78.7%) and lower mean survival time (44.94±27.47) than T1/2 cases (n=29, 100% and 55.00±10, 77) (*p*=0.011 and *p*=0.032, respectively). Most cases had stage III / IV (n = 60, 78.9%) and moderately differentiated carcinomas (n=65, 85.5%) (Table 1).

**Figure 1:**
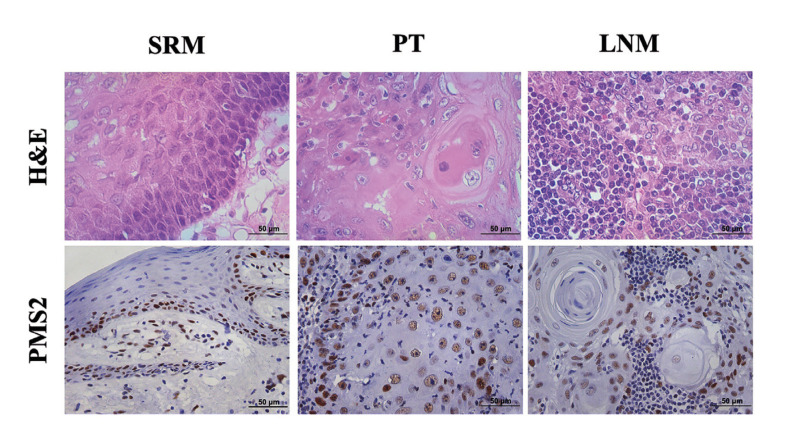
Histopathological and immunohistochemical profile of PMS2 immunostaining in surgical resection margins (SRM), OSCC primary tumor (PT) and OSCC lymph node metastasis (LNM). Magnification=400x; H&E and IHC.

Tumor, lymph node, and surgical resection margin tissues were evaluated. The immunoreactivity for these proteins was predominantly detected in cell nuclei. Positive immunoreaction for PMS2 was higher in tumors (59.7±28.6%, *p* <0.001) than in surgical resection margins (26,1±18,1%) and in lymph nodes (48,6±27,3%) (Fig. 1). PMS2 levels were also higher in tumors from other locations (mouth vestibule (n=8), alveolar ridge (n=4), retromolar area (n=2), gingiva (n=1)) than in floor of the mouth and tongue (*p*=0.006). Higher expression of PMS2 was found in N0/1 tumors compared to N2/3 (*p*=0.026). Comparison of histological grades revealed that patients with poorly differentiated carcinomas showed greater immunostaining for PMS2. (*p*=0.013) (Table 2).

**Figure 2:**
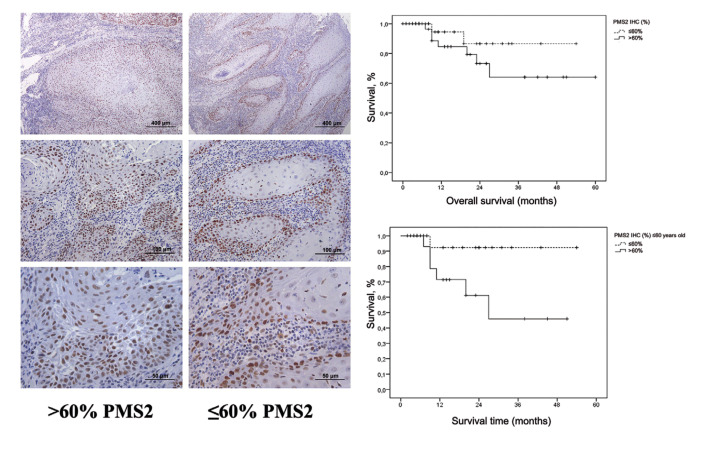
Immunohistochemical profile of high (>60%) and low (≤60%) PMS2 immunostaining in primary OSSC tumor and its influence on overall survival in all samples based on age (60 years).

Considering that the median positive immunostaining for PMS2 was 59.1% (range = 0.1 to 100.0), we adopted 60% as a cutoff point to define low and high expression of this immunomarker. The median overall survival time was 49±3 months, with 49±4 months for patients with low expression of PMS2 and 44 ±7 months for those with high expression (*p*=0.223) (Fig. 2).

In patients >60 years of age, immunostaining for PMS2 did not significantly influence overall survival (*p*=0.155) (Table 3), whereas in ≤60 years old patients (22; 95.6%; 50.4±3.3 months) a significant association was observed with lower survival (*p*=0.029), with PMS2 immunostaining reaching >60% (*p*=0.041) (Fig. 2) (Table 3).

**Table 2:**
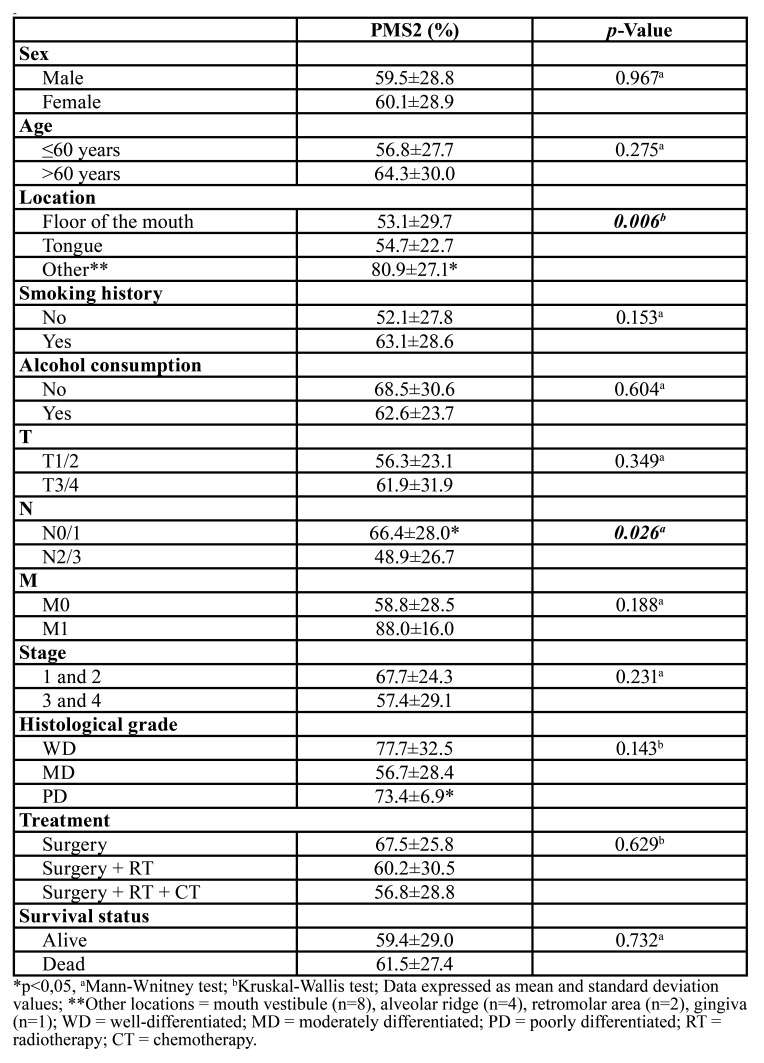
Influence of the clinical-epidemiological profile of the selected patients on the immunoexpression of PMS2.

**Table 3:**
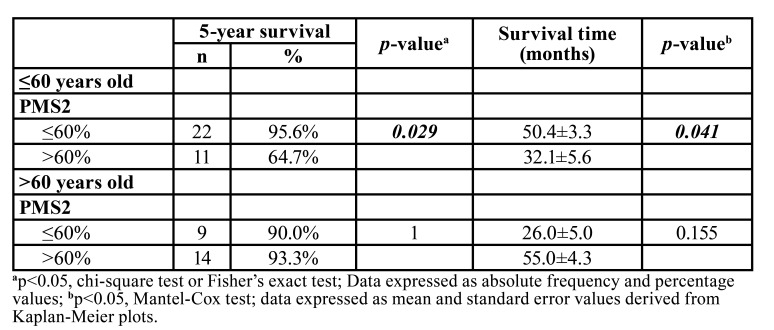
Influence of the PSM2 immunoexpression on the frequency of death of patients under and over the age of 60 years treated at the Haroldo Juaçaba Hospital (Cancer Institute of Ceará).

In multivariate analysis the factors of univariate analysis did not show significant influence on the overall survival of the sample. In ≤60 years old patients, treatment (*p* = 0.030) and high PMS2 immunostaining (*p* = 0.042) significantly increased the hazard risk of death (7.69 and 5.36 times), whereas in patients >60 years of age these variables did not influence the overall survival (Table 4).

**Table 4:**
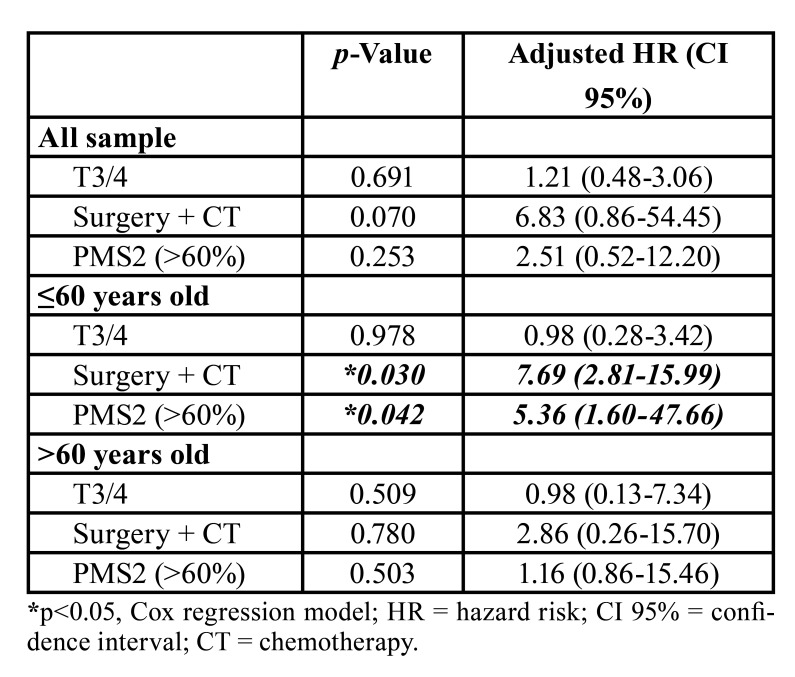
Multivariate analysis of risk factors to overall survival in patients under and over the age of 60 years treated at the Hospital Haroldo Juaçaba (Cancer Institute of Ceará).

## Discussion

The development of OSSC is highly associated with environmental and lifestyle factors, which highlights the role of epigenetic factors in oral carcinogenesis (18). The relationship between immunostaining of MMR proteins and cancer has been studied in the literature, with solid findings related to CRC (11,19). An imbalance caused by overexpression of MMR proteins was also observed in non-small cell lung cancers (12), prostate cancer (14), and gastric cancers (20). However, there are only a few studies correlating the MMR pathway with OSCC, and the results are still controversial (3,21).

The clinical-pathological data of our study showed that most patients were male and had tumors located on the floor of the mouth and tongue, which is in accordance with several studies (9,22), thus exhibiting classic features of this disease. In the present investigation, only tumor size influenced the 5-year survival. This finding has also been demonstrated in a study of an Indian population sample, in which patients with T4 tumors remained alive for less than 5 years (23). In agreement with the current literature, our work indicated that stage T1/2 tumors were associated with significantly higher survival (1,24).

When correlating the overexpression of PMS2 with clinical and pathological characteristics, an association with tumors from other locations and lymph node stage (N0/1) was detected. Similar association with lymph nodes was also found in a study investigating microsatellite instability (MSI) in gastric cancer, in which tumors with MSI showed a higher rate of N0 stage than sTable microsatellite tumors (13).

The overexpression of MMR proteins could be a compensatory mechanism to a deficient functional performance (1), which highlights the theory of a hypomorphic PMS2 variant (11). Previous studies detected reduced MMR protein expression during carcinogenesis (7,9,18,22). However, these studies focused on the carcinogenic process of potentially malignant lesions.

In contrast, studies with mammalian cells demonstrated that the overexpression of either wild-type or truncated hPMS2 results in an insTable genomic phenotype, similar to the process observed in MMR deficiency (15,25).

In our investigation, high levels of PMS2 were linked to lower survival. These results corroborate previous findings demonstrating that the overexpression of only one component of the MMR complex was capable of interrupting the adequate function of the MMR pathway (15,25). This could contribute to genetic instability, increasing the risk of carcinogenesis and cancer progression. A similar effect was also found in the MutSa complex (MSH2/MSH6) in patients with OSSC (1). Higher expression of MSH6 was associated with poor prognosis, whereas MSH2 expression had no impact on this variable. However, the analysis of both proteins combined revealed that high expression of the MutSa complex was an independent prognostic factor for poor overall survival (1).

In our study, in patients 60 years of age and younger high levels of PMS2 led to a worse prognosis. A large cohort of 748 HNC cases demonstrated that younger patients with OSCC had worse outcomes compared to patients with non-oral cavity cancers (4). Moreover, in the same study, non-smoking/non-drinker patients with OSCC were examined under the hypothesis of human papillomavirus (HPV) influence (4). However, no positive correlation was found, as HPV is more often correlated with oropharyngeal squamous cells carcinoma (26). Goodengerb *et al*. (11) suggested that an early onset of cancer could be observed in PMS2 mutation carriers. These findings support the theory that genetic factors may be contributing to the development and progression of these tumors in patients not exposed to classic risk factors.

Our data also suggests that younger patients treated with surgery combined with chemotherapy have poorer survival. This finding has significant implications from the treatment perspective. These patients commonly receive platinum-based chemotherapy which require a functional MMR system to induce cell apoptosis in response to DNA damage caused by the treatment (3,27). A study using a subcutaneous xenograft mouse model with prostate cancer cells lacking PMS2 protein (DU145) demonstrated that PMS2 played a role as a tumor suppressor by increasing apoptosis upon its introduction. Nevertheless, the overexpression of PMS2 as a compensatory mechanism can disrupt the cytotoxic signaling pathway. This leads to non-productive interactions with pro-apoptotic factors, thus enhancing tolerance to DNA damage (15).

The retrospective nature of this unicentric study could limit its level of clinical evidence. Nonetheless, our findings indicate that, in younger patients, higher levels of PMS2 and treatment consisting of surgery combined with chemotherapy significantly increased the risk of death. Thus, this study is a relevant addition to the limited available data as it could guide future genetic and epidemiologic investigations focusing on the role of the MMR proteins in OSCC.
